# Structural mechanism of outer kinetochore Dam1:Ndc80 complex assembly on microtubules

**DOI:** 10.1126/science.adj8736

**Published:** 2023-12-07

**Authors:** Kyle W. Muir, Christopher Batters, Tom Dendooven, Jing Yang, Ziguo Zhang, Alister Burt, David Barford

**Affiliations:** 1 MRC Laboratory of Molecular Biology; Francis Crick Avenue, Cambridge, CB2 0QH, UK

## Abstract

Kinetochores couple chromosomes to the mitotic spindle to segregate the genome during cell division. An error correction mechanism drives the turnover of kinetochore – microtubule attachments until biorientation is achieved. The structural basis for how kinetochore-mediated chromosome segregation is accomplished and regulated remains an outstanding question. Here we describe the cryo-electron microscopy structure of the budding yeast outer kinetochore Ndc80 and Dam1 ring complexes assembled onto microtubules. Complex assembly occurs through multiple interfaces, and a staple within Dam1 aids ring assembly. Perturbation of key interfaces suppresses yeast viability. Force-rupture assays indicated this is a consequence of impaired kinetochore – microtubule attachment. The presence of error correction phosphorylation sites at Ndc80-Dam1 ring complex interfaces and the Dam1 staple explains how kinetochore – microtubule attachments are destabilized and reset.

Chromosome segregation is essential for the equal propagation of genetic information from parent to daughter cells, achieved through kinetochore-mediated coupling of sister chromatids to the mitotic spindle (1). Kinetochores are large macromolecular assemblies delineated into the inner – centromere binding- and outer -microtubule binding- complexes. The outer kinetochore couples the inner kinetochore CCAN (constitutive centromere associated network) to microtubules through three conserved complexes comprising the Knl1-MIND-Ndc80 (KMN) network (2). MIND interconnects CCAN with both the Ndc80 and Knl1 complexes (Ndc80c and Knl1c). Knl1c functions in the spindle-assembly checkpoint (SAC) (3), whereas Ndc80c, a heterotetramer of Ndc80, Nuf2, Spc24 and Spc25, is the major microtubule-binding component of KMN.

Outer kinetochore attachment to microtubules is augmented by additional essential microtubule-dependent kinetochore components. Many fungi, including *S. cerevisiae*, utilize the ten-subunit Dam1 complex (Dam1c), which self-assembles into a large ring around microtubules (4–9), whereas most metazoans contain the Ska complex (2). Although the Ndc80c and Dam1/Ska complexes bind microtubules independently, full function and interaction strength requires both modules (10–17). A central unresolved question is how these complexes coordinately bind and track the dynamic microtubule plus-end to ensure kinetochores do not detach from the spindle (10, 12, 15, 16, 18, 19). As initial kinetochore-microtubule attachments are not necessarily bioriented, an error correction (EC) pathway, governed by antagonistic outer kinetochore phosphorylation by Aurora B kinase, ensures erroneous attachments are weakened and reset (20–24). As kinetochores come under tension, a feature of biorientation, the outer kinetochore is dephosphorylated, attachments are stabilized, and the SAC is inactivated to trigger anaphase (20, 21, 23, 25–31).

In yeast, centromere-localized Aurora B kinase phosphorylates Dam1c to suppress ring formation (15, 17, 32) and block its association with Ndc80c (10, 11, 28, 33), resulting in weakened end-on attachment (34, 35). Phosphorylation of a second set of targets within the unstructured N-terminus of Ndc80 (Ndc80^N-Tail^) also contributes to EC (14, 35, 36).

To obtain deeper mechanistic insight into Dam1c:Ndc80c (outer kinetochore^Dam1c:Ndc80c^) assembly on microtubules and its regulation by EC, we reconstituted and determined the cryo-electron microscopy (cryo-EM) structure of the budding yeast outer kinetochore^Dam1c:Ndc80c^:microtubule complex. We observe multiple contacts between Ndc80c and Dam1c. An N-terminal ‘staple’ region of the Dam1 subunit (Dam1^Staple^) binds the inter-protomer interface of the Dam1c ring. The presence of embedded EC phosphorylation sites within the Dam1^Staple^ and Ndc80c-Dam1c interfaces indicates why EC would drive both Dam1c ring disassembly and destabilize kinetochore-microtubule attachments.

## Results

### Overall architecture of the yeast outer kinetochore^Dam1c:Ndc80c^ bound to microtubules

**Figure 1:**
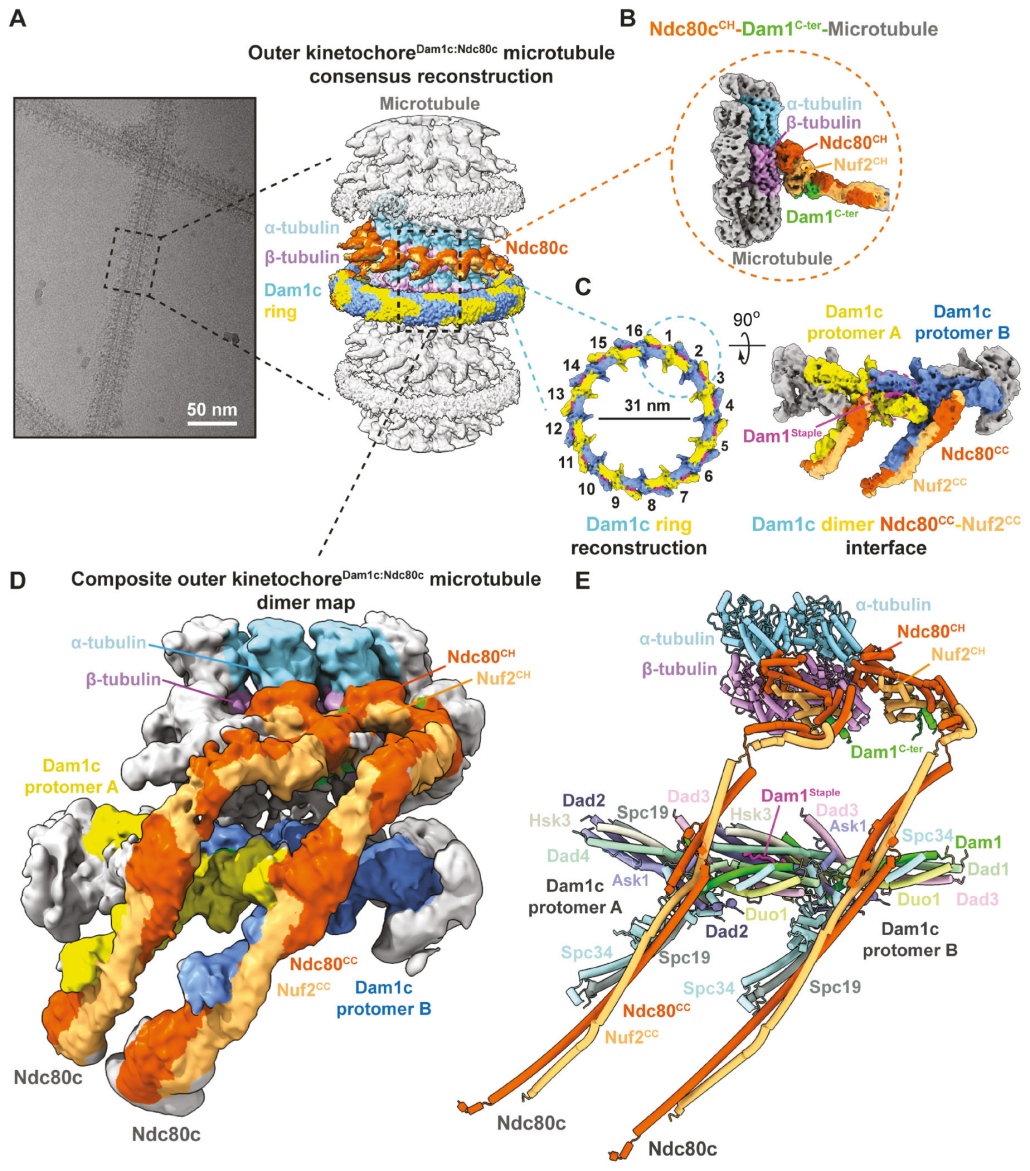
Yeast outer kinetochore^Dam1c:Ndc80c^ complexes assemble through multiple interfaces to tandemly decorate the microtubule. **(A)** Representative cryo-EM micrograph and corresponding consensus reconstruction of the outer kinetochore^Dam1c:Ndc80c^-microtubule complex. **(B)** Cryo-EM density map of the Ndc80c^CH^-Dam1^C-ter^-microtubule reconstruction. **(C)** Cryo-EM reconstruction of the 16-subunit Dam1c^Ring^, and symmetry expanded Dam1c dimer Ndc80^CC^:Nuf2^CC^ interface. **(D)** Composite cryo-EM map and corresponding atomic model **(E)** of a yeast outer kinetochore^Dam1c:Ndc80c^:microtubule dimer (defined as two copies of α/β tubulin:Dam1c:Ndc80c). Dam1c^Ring^ augments outer kinetochore^Dam1c:Ndc80c^ tracking of both polymerizing and depolymerizing microtubule tips (11, 15, 16). The structure suggests Ndc80c is borne along as a ‘passenger’ on the outer surface of the ring, and need not necessarily bind the microtubule. In the latter, the complex is directly pushed by the ring.

Outer kinetochore^Dam1c:Ndc80c^:microtubule complexes were assembled on cryo-EM grids by step-wise addition of taxol-stabilized microtubules, Ndc80c, and Dam1c (Fig. 1A, fig. S1A). Cryo-electron micrographs showed microtubules decorated with Dam1c rings (Dam1c^Ring^) and Ndc80c fibrils (Fig. 1A). In the resultant consensus cryo-EM map, we observed a tandem organization of Ndc80c coiled-coil ‘spokes’ emerging from the microtubule surface between diffuse Dam1c rings (Fig. 1A, fig. S2A, B).

The intermediate resolution of the cryo-EM map and the incompatible symmetries between the 16 protomers of the Dam1c^Ring^ and the 13 protofilament microtubule necessitated a divide and consolidate strategy for structure determination **(Materials and Methods**). This approach generated reconstructions of Ndc80c, and a dimer of Dam1c protomers at resolutions of 3.5 Å and 3.15 Å, respectively (Fig. 1B-D, figs S2B, C, S3A, S4, Table S1). Cryo-EM density was not visible for the Spc24:Spc25 subunits of Ndc80c. The coiled-coils of Ndc80c emanate from the calponin homology (CH) domains of the Ndc80 and Nuf2 subunits (Ndc80^CH^ and Nuf2^CH^) at the surface of the microtubule to fold across the outer surface of the Dam1c^Ring^. Here we observed prominent density corresponding to the coiled-coil central region of the Ndc80:Nuf2 subunits (Fig. 1C, D, fig. S2C). To obtain a complete model of the complex, we generated a composite map comprising two copies of the kinetochore^Dam1c:Ndc80c^ protomer unit by rigid body fitting of the separate volumes into the consensus outer kinetochore^Dam1c:Ndc80c^:microtubule cryo-EM map (Fig. 1D). We then ‘folded’ the Ndc80:Nuf2 coils in the Dam1c^Protomer^ component of the map until they were in proximity with the coils emanating from the Ndc80c^CH^ domains, and melded the models to generate a structural model of the yeast kinetochore^Dam1c:Ndc80c^:microtubule complex (Fig. 1D, E, Table S3, Movie S1).

### Structure and regulation of Dam1c^Ring^ assembly by a Dam1 staple peptide

Two- and three-dimensional classification showed that the Dam1c^Ring^ is tilted at variable angles relative to the microtubule (fig. S2B,C, **top left panel**), similar to Dam1c^Ring^ alone (8). Consistently, the tubulin surface beneath the ring is unoccupied except for diffuse density emerging from the α/β-tubulin C-terminal tails (fig. S3B), potentially representing the acidic E-hooks of α- and β-tubulin that enhance microtubule – Dam1c interactions (5, 8). The Dam1c^Ring^ – microtubule interface potentially involves flexible participants, as supported by biochemical and crosslinking mass spectrometry data (7, 11, 32, 37).

**Figure 2:**
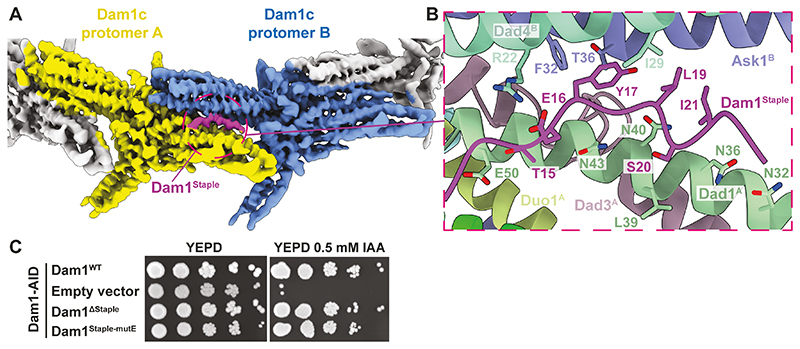
The Dam1 N-terminus forms a staple to stabilize Dam1c^Ring^ assembly that is negatively regulated by Aurora B kinase. **(A)** Structure of the Dam1c protomer dimer interface shows a staple density (purple) between protomer A (yellow) and protomer B (blue). (**B**) Details of amino-acid contacts at the Dam1c^Protomer^ dimer – Dam1^Staple^ interface. Ser20 is an Aurora B kinase phosphorylation site and is oriented toward the Dad1 subunit of protomer A. Dam1^Staple^ residues 13-23 are visible in cryo-EM density, whereas residues 1-12, and the connectivity to residue 55 of the remainder of Dam1 are not resolved. Mutation of Dad1 Glu50 to Asp impairs ring formation (41). **(C)** Dam1 auxin depletion assays. Cells grown on YEPD agar and YEPD agar containing 0.5 mM IAA are shown for each strain. Dam1^ΔStaple^: Dam1 mutant with residues 1-26 deleted, Dam1^Staple-mutE^: Dam1 mutant: Y17E/L19E/I21E.

Dam1c^Ring^ assembly follows a shoulder-to-shoulder configuration (7, 38) (Figs 1C, E and 2A). The inter-protomer junctions are similar to the cryo-EM structure of a truncated *C. thermophilum* Dam1c^Ring^ (38). In this structure, the map resolution is limited to 4.5 Å. In contrast, here we find that in all ten subunits of *S. cerevisiae* Dam1c most side-chains within the globular core of the complex are resolved (fig. S3C). Additionally, we resolved a ‘staple’ density located at the inter-Dam1c^Protomer^ interfaces that was truncated from *C. thermophilum* Dam1 (38) (Fig. 2A, B). The Dam1^Staple^ comprises the N-terminus (residues 1-26) of the Dam1 subunit. The staple bridges the Dam1 and Dad1 subunits of Dam1c^ProtomerA^ with the Ask1 and Dad4 subunits of Dam1c^ProtomerB^. Contact with Dam1c^ProtomerA^ is through hydrogen bonding between Dam1^Staple^ residues Thr15 and Ser20, and Dad1^A^ (Fig. 2B). Binding to Dam1c^ProtomerB^ is mediated by a salt bridge between Arg22 of Dad4^B^ and Glu16 of Dam1^Staple^, as well as packing of Dam1^Staple^ residues Tyr17, Leu19, and Ile21 against Ask1^B^ and Dad4^B^ (Fig. 2B). To test this interface in vivo, we performed rescue assays in an *S. cerevisiae* strain with an auxin-inducible degron (AID) tag inserted at the C-terminus of the endogenous copy of Dam1 (Table S2). We then integrated a series of Dam1 variants at the *LEU* locus. Cells grown on auxin with wild-type Dam1 as the ectopic copy rescued loss of endogenous Dam1, whereas cells carrying an empty vector failed to grow (Fig. 2C, fig. S1B, C). Cells carrying either a Dam1^ΔStaple^ or Dam1^Staple-mutE^ (Dam1 Tyr17, Leu19 and Ile21 to Glu) mutant were viable in the absence of wild-type Dam1 (Fig. 2C). In the cell, Dam1c^Ring^ assembly may be augmented by additional mechanisms that compensate for mutagenesis of the Dam1^Staple^ (39, 40).

Serine 20 of Dam1^Staple^, a target of Aurora B kinase (28), is buried at the interprotomer interface close to Leu39, Asn40 and Asn43 of the Dad1 subunit of Dam1c^ProtomerA^ (Fig. 2B).

Phosphorylation of Ser20 would cause steric hindrance and charge repulsion that would disrupt Dam1^Staple^ binding. Phosphorylation of Ser20 destabilizes Dam1c^Ring^ in vitro (17, 32), and increases its diffusion on microtubules (12, 15). Similarly, mutations at the Dam1^Staple^-binding site (Fig. 2B) impair ring formation on microtubules (41). However, these mutations rescue loss of Aurora B kinase activity, presumably by driving increased kinetochore-microtubule attachment turnover (41, 42). Consistent with the notion that Dam1^Staple^ regulates ring assembly, we found that the assembly of higher-order Dam1 complexes is substantially impaired when the staple is deleted (fig. S1F, S3D, E).

### Structure of the Ndc80c:microtubule interface

**Figure 3:**
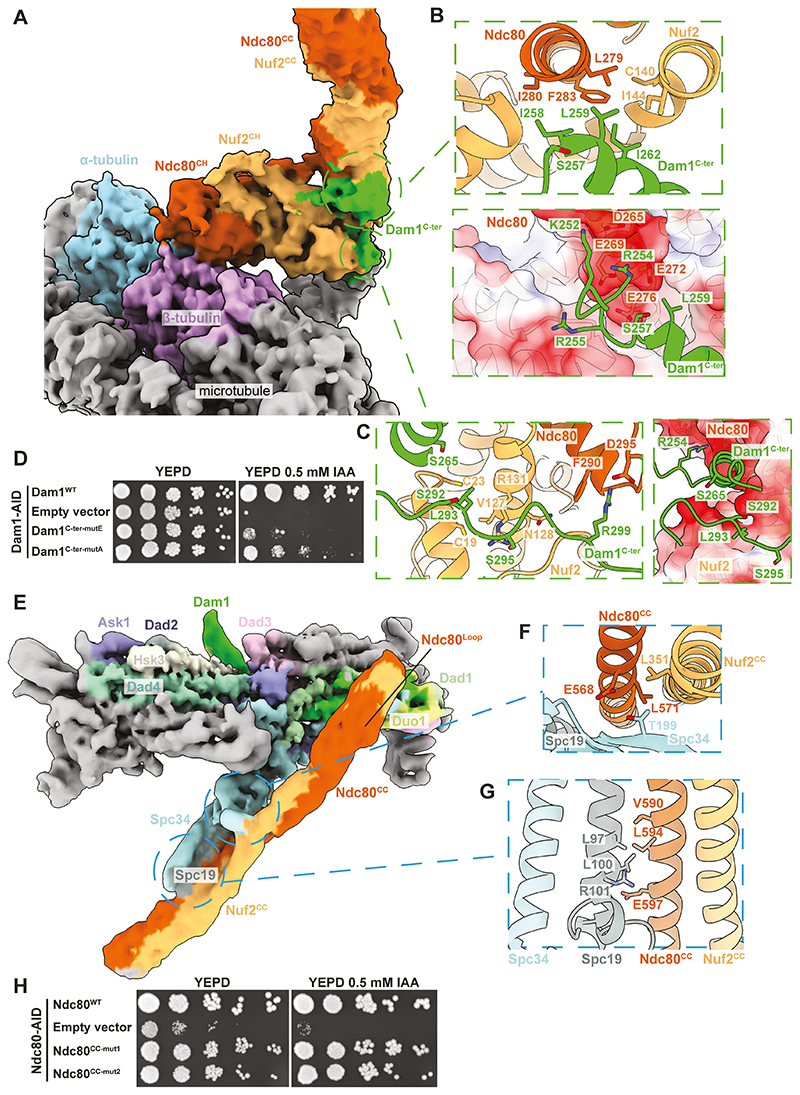
The Dam1 and Ndc80 complexes associate through a microtubule-proximal interface, and the outer surface of the Dam1c^Ring^. **(A)** Cryo-EM reconstruction of the Ndc80c-Dam1c-microtubule interface. Rigid-body placement of an AlphaFold2 prediction of the Ndc80c^CH^ – Dam1^C-ter^ interface into cryo-EM density shows: **(B)** The Dam1^C-ter^ α-helix packs against a hydrophobic interface generated by the Ndc80:Nuf2 coiled-coil emerging from the Ndc80c^CH^ domain (upper panel). Surface charges on Ndc80c^CH^ and position of the Ser257 EC site on Dam1^C-ter^ are shown in the lower panel. **(C)** The Dam1^C-ter^ interface on the back-side of the Ndc80c^CH^ (left panel), positions of residues targeted by EC and surface charges (right panel). Ser265 and Ser292 are oriented toward negative charges that would drive dissociation of Dam1^C-ter^ from Ndc80^CH^ during EC. **(D)** Dam1 rescue assays on auxin. Cells grown on YEPD agar and YEPD agar containing 0.5 mM IAA are shown for each strain. Dam1^C-ter-mutE^: Dam1 mutant: I258E/L259E/I262E, Dam1^C-ter-mutA^: Dam1 mutant: I258A/L259A/I262A. **(E)** Cryo-EM reconstruction of the Dam1c monomer-Ndc80:Nuf2 coiled-coil interface. The position of the Ndc80^Loop^ is highlighted in fig. S5D. Rigid-body placement of an AlphaFold2 prediction of the Ndc80^CC^:Nuf2^CC^ into cryo-EM density together with our experimental model of Dam1c shows the Ndc80^CC^:Nuf2^CC^ binds Dam1c at two interfaces shown as insets: **(F)** Ndc80^CC^:Nuf2^CC^ docks against a β-strand on Spc34. The EC target site Thr199 is oriented toward Ndc80c. **(G)** The Spc19:Spc34 coiled coils pack against Ndc80^CC^:Nuf2^CC^ via residues in Spc19 and Ndc80. **(H)** Ndc80 auxin depletion assays. Cells grown on YEPD agar and YEPD agar containing 0.5 mM IAA are shown for each strain. Ndc80^cc-mut1^: Ndc80 coiled-coil mutant1: E568A/V590A/L594A/E597A, Ndc80^cc-mut2^: Ndc80 coiled-coil mutant2: E568R/V590W/L594E/E597R.

The Ndc80c microtubule-binding domain forms a club-like structure comprising its two CH domains (Figs 1B, E and 3A). The underside of the club binds the α/β-tubulin interface along the lateral axis of the protofilament, whereas the coiled-coil shaft of Ndc80:Nuf2 projects outward orthogonal to the CH domains. In contrast to human and *C. elegans*, in which Ndc80c binds to every lateral α/β-tubulin interface (43, 44), the yeast complex binds only at the α/β site, a mode of binding that is independent of Dam1c (fig. S5A-C). No EM density is visible for the disordered Ndc80^N-tail^, and therefore we cannot account for how it contributes to kinetochore – microtubule attachments.

### Binding of the Dam1 C-terminus to Ndc80c is essential and is regulated by error correction

We observed additional cryo-EM density at the base of the Ndc80:Nuf2 coiled-coils emerging from Ndc80c^CH^ (Figs 1B and 3A). Although the additional density was not sufficiently well resolved to model *de novo*, crosslinking mass-spectrometry and mutagenesis experiments suggest this region of Ndc80c (Fig. S5A) interacts with Dam1c (13) through the Dam1 C-terminus (11, 14). We used AlphaFold2 to predict the structure of Ndc80:Nuf2 together with residues 201 to 343 of Dam1. The resulting prediction was a tripartite complex consistent with a subsequently-solved crystal structure (45). Two short segments from the C-terminus of Dam1 (Dam1^C-ter^) dock onto the Ndc80c^CH^ domains. One of these (residues 251 to 272) nestles in an amphipathic pocket at the base of the Ndc80:Nuf2 coiled-coils (Fig. 3B, fig. S5D), and another (residues 287 to 301) snakes around the back-face of Ndc80c^CH^ (Fig. 3C, fig. S5E). Dam1 residues Ile258, Leu259, Ile262 pack against Ndc80c^CH^ (Fig. 3B, **upper panel**). We introduced Dam1 mutants bearing triple alanine or glutamate substitution of these residues as ectopic copies into our Dam1-AID strain (Dam1^C-ter-mutA^ and Dam1^C-ter-mutE^, respectively) (figs S1B, C, Table S2). Upon depletion of endogenous Dam1, growth was severely impaired (Fig. 3D). Therefore, the integrity of the Dam1^C-ter^:Ndc80c^CH^ interface is essential for proper kinetochore function and yeast cell viability.

During EC, residues at the Dam1^C-ter^-Ndc80c^CH^ interface are phosphorylated by Aurora B kinase, specifically Dam1 Ser257, Ser265, and Ser292 (Fig. 3B-lower panel, 3C-right panel) (27, 28). Phosphorylation of these residues in vitro results in decreased strength and lifetime of outer kinetochore^Dam1c:Ndc80c^ binding to microtubules (10, 11), and phospho-mimetics suppress impaired Aurora B kinase activity in vivo (28). Ser257 is oriented towards negatively charged residues on an α-helix of Ndc80c (Fig. 3B, **lower panel**). Phosphorylation of Ser257 would be incompatible with binding of Dam1^C-ter^ to Ndc80c^CH^. Similarly, phosphorylated Ser265 and Ser292 would also be oriented toward negatively charged surfaces on Ndc80c (Fig. 3C, **right panel**). EC phosphorylation would thus weaken co-assembly of the outer kinetochore.

### The central coiled-coil domain of Ndc80:Nuf2 folds across the outer rim of Dam1c

The Ndc80:Nuf2 central coiled-coil docks near the Dam1c^Protomer^ interface against a pair of β-strands from Spc34 that contain the EC target residue Thr199 (Fig. 3E, F, **fig. SF left panel**), and runs parallel with the C-terminal coiled-coils of Spc34 and Spc19 (Fig. 3E, G, **fig. SF right panel**). Our structure is consistent with previous cross-linking mass spectrometry and insertion mutagenesis experiments indicating that the central segment of the Ndc80:Nuf2 coiled-coils proximal to the conserved Ndc80 loop directly interacts with the Spc34:Spc19 C-termini (11, 13). Following focused classification and flexible refinement, a distinct interruption in the coiled-coil became apparent (Fig. 3E). This interruption corresponds to the Ndc80 loop (Ndc80^Loop^), enabling us to infer the amino-acid register of an AlphaFold2 prediction comprising the remaining coiled-coil (fig. S5G). We mapped sequence conservation onto the structure, and observed that interfacial residues are well-conserved, whereas outward-facing residues are not (fig. S5H).

To test the impact of mutating this interface in vivo, we generated a yeast strain in which the endogenous copy of Ndc80 bore a C-terminal AID tag (figs S1D, E, Table S2). Mutation of a series of four conserved interfacial residues on Ndc80 (Ndc80^CC-mut1&2^) did not impair viability (Fig. 3H), consistent with in vitro observations that disruption of this interface by phosphorylation of Spc34 at Thr199 exerts a minor reduction in outer kinetochore – microtubule rupture forces (10). Indeed, our auxin-depletion experiments that disrupted the Dam1^C-ter^-Ndc80c^CH^ interface (Fig. 3D) showed that the Ndc80c coiled-coil:Spc34 interface is not sufficient for proper outer kinetochore function (13, 14).

### Disruption of outer kinetochore^Dam1c:Ndc80c^ interfaces weakens microtubule attachments in vitro

The full load-bearing potential of the outer kinetochore and its ability to track dynamic microtubule ends is dependent on co-assembly of Ndc80c and Dam1c (10–17, 33). Disruption of coordinated binding to the microtubule is a central proposed mechanism for how error correction regulates attachments (10–13, 15, 16, 33–35, 46).

**Figure 4:**
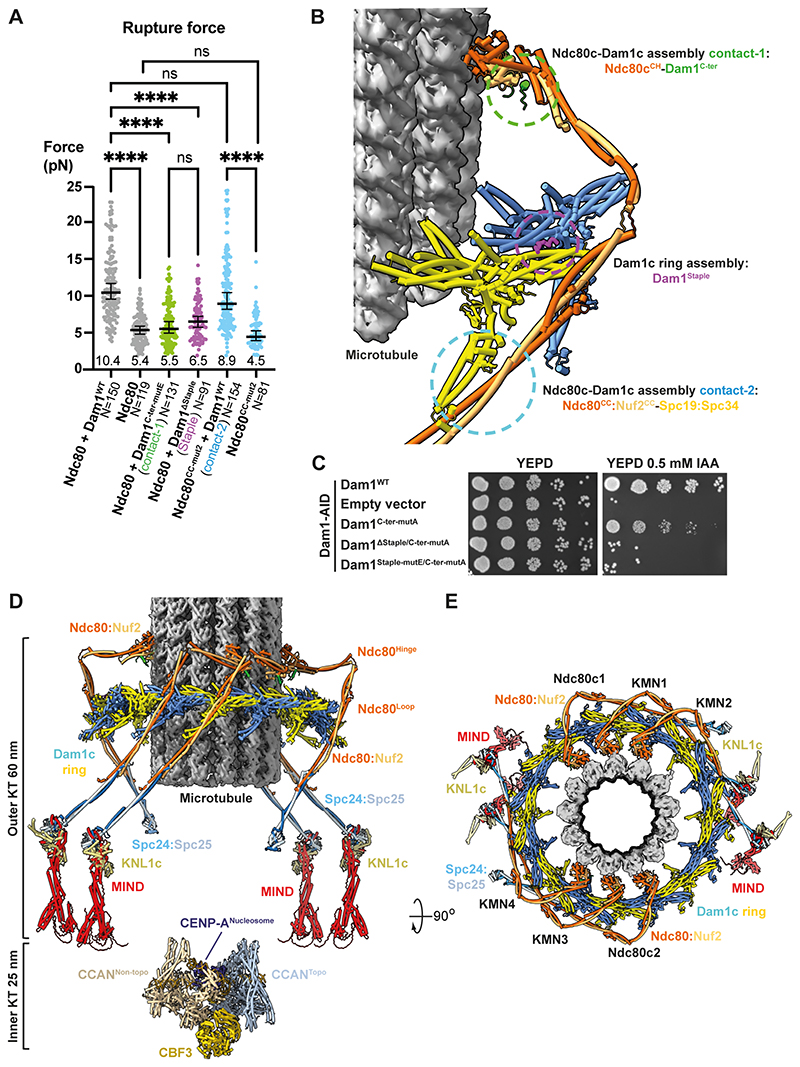
A structural model for mitotic error correction and chromosome segregation by the yeast kinetochore. **(A)** Force-rupture measurements of outer kinetochore – microtubule complexes. Each circle represents a single rupture event (the maximum trap force before rupturing). The total number of measurements for each condition are indicated by N values. The black bar represents the median rupture forces, with 95% CIs. Numbers below the black bar indicates median values. A Kruskal-Wallis test to determine which medians are significantly different was performed, ****: p<0.0001, ns: not significant. Mutants of Dam1 and Ndc80 are defined in Figs 2C, 3D, H. **(B)** Structure of an outer kinetochore^Dam1:Ndc80c^ dimer bound to the microtubule. Outer kinetochore and Dam1c^Ring^ assembly contacts that are targeted by error correction are highlighted. **(C)** Dam1 auxin depletion assays. Cells grown on YEPD agar and YEPD agar containing 0.5 mM IAA are shown for each strain. Dam1^C-ter-mutA^: Dam1 mutant: I258A/L259A/I262A, Dam1^ΔStaple/C-ter-mut-A^: Dam1 mutant with residues 1-26 deleted/I258A/L259A/I262A. Dam1^Staple-mutE/C-ter-mut-A^: Dam1 mutant: Y17E/L19E/I21E/I258A/L259A/I262A. Simultaneous mutation of the Dam1 Staple (Dam1^ΔStaple^ and Dam1^Staple-mutE^) and Dam1 C-terminus (Dam1^C-ter-mutA^) results in lethality. **(D)** Structural model of the complete yeast holo-kinetochore bound to a microtubule. Shown are the microtubule, Dam1c^Ring^, four KMN network complexes, two Ndc80 complexes and the CCAN inner kinetochore complex (comprising two CCAN promoters, CBF1, CBF3 and CENP-A nucleosome) (49). The flexible linkers connecting CCAN to the KMN network and Ndc80c are not shown. For clarity, shown are four of six possible KMN network complexes. The view is in plane with the microtubule helical axis. In this configuration, the end-to-end dimension of the kinetochore is ~85 nm, a value consistent with the relative location and separation of kinetochore components measured in metaphase yeast cells using fluorescence localization microscopy (52, 62). Shortening of the kinetochore as tension is reduced (52) could be facilitated through the flexible linkers connecting the inner kinetochore with MIND and Ndc80 complexes. **(E)** Top view viewed from the microtubule minus-end.

In vitro optical trap experiments in which purified yeast kinetochores and reconstituted kinetochore – microtubule attachments are challenged have determined rupture forces of ~5-10 pN (10, 12, 15, 47). To test the contribution each of the interfaces in our structure makes to the ability of the outer kinetochore^Dam1c:Ndc80c^ complex to withstand force, we purified a series of Dam1c and Ndc80c variants (figs S1F-H) and performed force-rupture assays (Fig. 4A, B, fig. S6) (10, 48). We determined a median rupture force of 5.4 pN for Ndc80c alone that increased to 10.4 pN in the presence of Dam1c (Fig. 4A), confirming that the kinetochore^Dam1c:Ndc80c^ complex withstands greater forces than does isolated Ndc80c (10, 12, 15). Mutation of the Dam1^C-ter^ (Ndc80c^CH^ contact-1) and Dam1^Staple^ (ring assembly contact) substantially lowered the rupture forces to 5.5 pN and 6.5 pN, respectively. The Dam1^C-ter^ mutant was not impaired in self-assembly (fig. S1I), consistent with (5), thus it presumably acts by disrupting binding to Ndc80c^CH^. In contrast, the Ndc80^CC-mut^2 (Spc19:Spc34-Ndc80:Nuf2 coiled-coil contact-2) essentially had no effect on rupture strength when combined with Dam1c (Fig. 4A, B). Hence, mutation of outer kinetochore assembly through the Dam1^C-ter^-Ndc80c^CH^ interface, or disruption of Dam1c^Ring^ formation independently weakened microtubule binding. Our structural and biochemical results suggested that ablation of these functional elements of Dam1, targeted for inhibition by EC, would generate complexes that are unable to assemble rings, contact Ndc80c, or bear load (Fig. 4A, B). To test whether concurrent disruption of these elements of Dam1c impacted kinetochore function in vivo, we combined Dam1^Staple^ and Dam1^C-ter^ mutants in our Dam1-AID strain (Table S2). Mutagenesis of key Dam1^C-ter^ residues to alanine (Dam1^C-ter-mutA^) permitted weak growth that was completely abolished when the Dam1^Staple^ was deleted or mutated (Dam1^ΔStaple^ and Dam1^Staple-mutE^, respectively) (Fig. 4C).

Consistent with EC driving attachment turnover by breaking outer kinetochore assembly, our force-rupture data correspond well to measurements of in vitro reconstituted Dam1c phosphorylated at the respective interfaces by Aurora B (10, 12, 15), as well as for complexes containing a phospho-mimetic Dam1 S20D mutation (12, 34). Finally, the severity of the force-rupture phenotypes correlated closely with the degree of viability defect caused by the corresponding mutants in cells. Mutagenesis of the interfaces identified in our structures likely caused reduced fitness in vivo because these cells form defective kinetochore – microtubule attachments that cannot support normal chromosome segregation.

## Discussion

We consolidated previous structural findings and in vivo measurements of relative kinetochore subunit positions and stoichiometry with our outer kinetochore^Dam1c:Ndc80c^ structure to generate a model for a yeast holo-kinetochore bound to a microtubule (Fig. 4D, E). The inner kinetochore generates a total of eight Ndc80c linkages to the microtubule (49). These Ndc80c molecules are readily accommodated by the 16 binding sites of Dam1c^Ring^, which is recruited through Ndc80c in a microtubule-dependent fashion (50). The flexible hinge between Ndc80c^CH^ and Ndc80^Loop^ (51) permits Ndc80 complexes to fold as hooks across the Dam1c^Ring^, explaining how the coiled-coils of Ndc80:Nuf2 dock in a parallel configuration across Spc19:Spc34 despite adherence of the Ndc80c^CH^ to the pseudo-helical symmetry of the microtubule (52, 53).

In budding yeast several models for how EC detects attachment errors have gained prominence (54). Yeast error correction is trifurcated between disruption of Dam1c^Ring^ assembly, disassembly of Dam1c – Ndc80c interfaces and phosphorylation of Ndc80^N-Tail^ (10–12, 14–16, 27, 28, 33). Collectively these inhibitory activities dismantle the Dam1c^Ring^ and dissolve kinetochore – microtubule attachments. We found that kinetochore^Dam1c:Ndc80c^ assembly is mediated through multiple interfaces that are antagonized by EC, whereas Dam1c^Ring^ assembly is facilitated by a ‘staple’ that contains an embedded EC target. In contrast to human Ndc80c, we did not observe inter-Ndc80c contacts, nor can we account for the flexible Ndc80^N-Tail^ (43, 55). The Dam1, Ask1 and Spc34 subunits of Dam1c form a network of interactions between the Dam1c^Ring^ and Ndc80c that promote cooperative outer kinetochore assembly on the microtubule and are disrupted by EC (10, 11, 13, 14). The region implicated in Ask1-Ndc80c binding is likely situated at the Ndc80c^Hinge^ and is too mobile to resolve in our cryo-EM maps (11). Our molecular models therefore account for two EC-sensitive contacts: the Dam1^C-ter^-Ndc80c^CH^ and the Spc34-Ndc80:Nuf2^CC^ interfaces. At both, residues phosphorylated in EC would cause electrostatic and steric repulsion (Figs 2B, 3B, C), explaining how EC-mediated phosphorylation suppresses kinetochore^Dam1c:Ndc80c^ assembly.

A fundamental paradox at the heart of EC is how initially weak, low-tension kinetochore attachments can escape re-phosphorylation during attachment reset to form new connections to the microtubule. Ndc80c retains a degree of microtubule affinity even when Ndc80^N-tail^ is either deleted (14, 56) or incorporates EC phosphosites (33). However, it has remained unclear how Dam1c is reincorporated into the kinetochore if the Ndc80c – Dam1c interfaces are disrupted during EC. The major targets of phosphorylation that govern kinetochore^Dam1c:Ndc80c^ assembly are within Dam1c (10, 11, 27, 28). Achieving full attachment strength would thus require dephosphorylation and/or replacement of Dam1c. We propose that simple replacement of Dam1c by EC-mediated turnover would resolve the EC-reset paradox. Following EC, kinetochores remain attached to the lateral face of the microtubule (35, 57), and phosphorylated Dam1c diffuses away. However, Dam1c not associated with the kinetochore remains unphosphorylated (29) and is transported to the kinetochore by tracking microtubule plus-ends. Unphosphorylated Dam1c then associates with Ndc80c to facilitate conversion from lateral to end-on attachment (33, 35, 57). This Ndc80c:Dam1c assembly could drive displacement of the outer kinetochore, rendering it resistant to the centromere-localized Aurora B kinase (58, 59).

Two models posit how the mechanical energy of microtubule depolymerization is exploited by the kinetochore to segregate chromosomes: the conformational wave, and biased diffusion. In the former, the curling protofilaments lever the kinetochore toward the spindle poles (60). In the biased diffusion model, an array of kinetochore attachments detaches and rebinds the tubulin lattice as it depolymerizes (61). Dam1c augments outer kinetochore tracking of both polymerizing and depolymerizing microtubule tips (11, 15, 16). Our findings support a model for kinetochore-mediated chromosome segregation wherein Dam1c^Ring^ acts as a topological sleeve that is pushed along by the curved protofilaments of the depolymerizing microtubule, driving Ndc80c translocation through steric occlusion. By generating multivalency through coordinating multiple Ndc80 complexes, Dam1c^Ring^ also enables biased diffusion. Association of outer kinetochore components through flexible peptidic interfaces mediates a tethering mechanism which prevents Ndc80c detaching from the microtubule when displaced by the advancing Dam1c^Ring^, thus pulling chromatids toward the spindle poles (6, 14–16, 33, 35).

## Supplementary Material

Movie S1

Supplementary Information

## Data Availability

Atomic coordinates and cryo-EM density maps (also listed in Table S1) have been deposited in the Protein Data Bank (www.rcsb.org) and the Electron Microscopy Data Bank (https://ebi.ac.uk/pdbe/emdb/): Ndc80c^CH^-α/β-tubulin-Dam1^C-ter^ (PDB: 8QAU; map: EMD-18304), Dam1c protomer dimer-Ndc80^CC^-Nuf2^CC^ (PDB: 8Q84; map: EMD-18246), Dam1c protomer monomer-Ndc80^CC^-Nuf2^CC^ (PDB: 8Q85; map: EMD-18247), Ndc80c – microtubule (map: EMD-18485). All other data are available in the main text or the Supplementary Materials.

## One-Sentence Summary

Structural mechanism for how phosphorylation regulates kinetochore-microtubule attachments in mitotic error correction.
